# No Evidence for a Role for Antibodies during Vaccination-Induced Enhancement of Porcine Reproductive and Respiratory Syndrome

**DOI:** 10.3390/v11090829

**Published:** 2019-09-06

**Authors:** Carmen A. Sautter, Ivan Trus, Hans Nauwynck, Artur Summerfield

**Affiliations:** 1 Institute of Virology and Immunology (IVI), Sensemattstrasse 293, 3147 Mittelhäusern, Switzerland; 2 Graduate School for Cellular and Biomedical Sciences, University of Bern, Freiestrasse 1, 3012 Bern, Switzerland; 3 Department of Infectious Diseases and Pathobiology (DIP), Vetsuisse Faculty, University of Bern, Länggassstrasse 122, 3012 Bern, Switzerland; 4 Laboratory of Virology, Department of Virology, Parasitology and Immunology, Faculty of Veterinary Medicine, Ghent University, Salisburylaan 133, 9820 Merelbeke, Belgium

**Keywords:** PRRSV-1, inactivated vaccine, homologous challenge, *in vivo*, antibody-dependent enhancement (ADE) of disease, monocyte-derived macrophages

## Abstract

Vaccination is one of the most important tools to protect pigs against infection with porcine reproductive and respiratory syndrome virus 1 (PRRSV-1). Although neutralizing antibodies are considered to represent an important mechanism of protective immunity, anti-PRRSV antibodies, in particular at subneutralizing concentrations, have also been reported to exacerbate PRRSV infection, probably through FcγR-mediated uptake of antibody-opsonized PRRSV, resulting in enhanced infection of, and replication in, target cells. Therefore, we investigated this pathway using sera from an animal experiment in which vaccine-mediated enhancement of clinical symptoms was observed. Three groups of six pigs were vaccinated with an inactivated PRRSV vaccine based on the PRRSV-1 subtype 3 strain Lena and challenged after a single or a prime-boost immunization protocol, or injected with PBS. We specifically tested if sera obtained from these animals can enhance macrophage infections, viral shedding, or cytokine release at different dilutions. Neither the presence of neutralizing antibodies nor general anti-PRRSV antibodies, mediated an enhanced infection, increased viral release or cytokine production by macrophages. Taken together, our data indicate that the exacerbated disease was not caused by antibodies.

## 1. Introduction

Antibody-dependent enhancement (ADE) of infection is a phenomenon that has been associated with many different viruses, such as lactate dehydrogenase elevating virus [1], HIV-1 [2,3], dengue virus [4,5], Japanese encephalitis virus (JEV) [6], and FMDV [7,8,9]. For some of these infections, this in vitro phenomenon has been associated with immunity-enhanced disease. A typical example of this is Dengue [5]. On the other hand, for other infections, even closely related ones such as JEV, no association of ADE antibodies with adverse vaccine effects or pre-existing immunity have been reported [6]. Typically, ADE was found at subneutralizing concentrations, at which antibodies are binding to the virus but are not sufficiently numerous to mediate neutralization. This binding facilitates viral uptake through FcRs, typically for IgG isotype antibodies. Alternatively, the antibodies can be specific for viral epitopes that are non-neutralizing. Theoretically, if the virus can replicate in FcR-expressing cells such as monocytes and macrophages, this would increase the viral replication and viral load, leading to more severe symptoms. Alternatively, or in addition, immune complexes can trigger an increased release of cytokines, which could potentially exacerbate symptoms [10,11] or indirectly downregulate an antiviral response [12,13]. This depends on the predominant antibody isotype and its preferential binding to either inhibitory or stimulatory FcRs.

ADE of disease in PRRSV was first described by Yoon et al., who investigated the enhancement of infection of alveolar macrophages and also found increased viremia and shedding of virus in vivo, after serum transfer to reach subneutralizing antibody titers [14]. They found that neutralizing antibodies with titers of 4 log_2_ were protective, but titers of 1 log_2_ increased viremia. In this study, the prototype PRRSV-2 strain VR-2332 was used. The same authors subsequently tested antibodies against this strain also for their ability to enhance the infection with heterologous strains and found a strain-dependent enhancement [15]. Further in vitro studies indicated the involvement of all three Fcγ receptors [16,17,18]. Using monoclonal antibodies, the Gp5/M complex was identified as a possible target for ADE antibodies [19].

Despite these studies, it is important to note that to our knowledge, enhanced disease after experimental vaccination with live attenuated vaccines (LAV) has not been observed, although the challenge infections were frequently performed in absence of neutralizing antibodies. There is also no indication that re-exposure with an antigenically distinct PRRSV strain that is not neutralized can result in enhanced disease [20,21,22].

However, in a vaccination challenge experiment, we found evidence for vaccine-induced enhanced disease for PRRSV. The pigs were vaccinated with an inactivated, homologous vaccine, followed by a challenge with the Lena strain. Lena represents a subtype 3 PRRSV-1 that emerged in Eastern Europe and stands out as a highly pathogenic PRRSV strain [23]. Following PRRSV challenge, some of the vaccinated animals developed more severe clinical symptoms and pneumonia, compared to mock-vaccinated animals. We, therefore, tested the sera from such animals for their ability to enhance infection of monocyte-derived macrophages (MDMs) and increase the amount of virus that would be released from the MDMs as well as to induce cytokine release. We used MDMs because they are a well-established cell culture model in PRRSV research, and allowed us to perform this research without having to sacrifice an unnecessarily large number of animals.

## 2. Materials and Methods

### 2.1. Animal Experiments

The vaccination experiments were performed at the Faculty of Veterinary Medicine at Ghent University, Merelbeke, Belgium. The local Ethical and Animal Welfare Committee of the Faculty of Veterinary Medicine of Ghent University approved the animal experiment (EC 2012/082, approval date: 11/07/2012).

**Figure A1:**
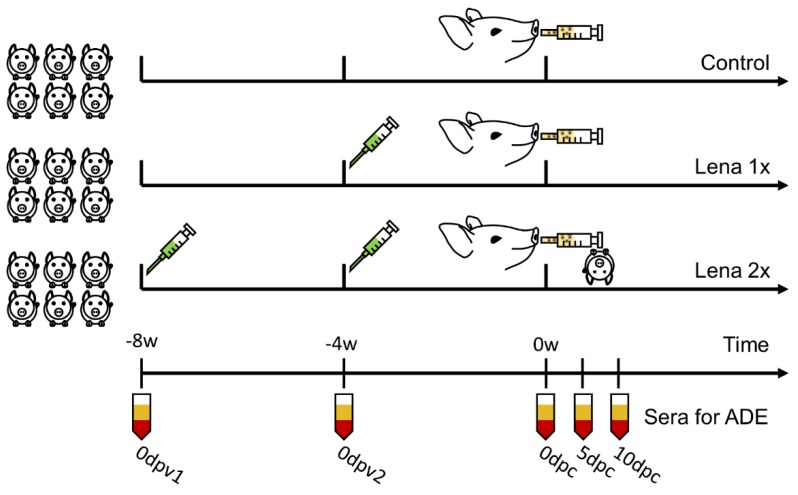
Experiment layout of vaccination and challenge.

Eighteen five-week-old pigs were obtained from a conventional, PRRSV-free farm in Belgium and tested to be free of PRRSV, porcine circovirus-2, and swine influenza virus by immunoperoxidase monolayer assay (IPMA). The piglets were randomly divided into three groups of six animals. The groups were either vaccinated once (“Lena 1x”), four weeks before the challenge, or twice (“Lena 2x”) at eight and four weeks before challenge (Appendix A, Figure A1). The age of the pigs at the time of the first immunization was six and ten weeks old. For the vaccination, binary ethyleneimine (BEI, Sigma-Aldrich, St. Louis, MO, USA)-inactivated Lena, grown on MARC-145 cells, formulated in commercial oil-in-water adjuvant (Suvaxyn, Zoetis Animal Health [24]; 1 mL/1 mL; 10^8^ TCID_50_/dose), was used. The pigs in the control group received 1 mL PBS with 1 mL adjuvant intramuscularly (i.m.) at eight and four weeks before the challenge. All animals were challenged by intranasal inoculation of 10^5^ TCID_50_ PRRSV Lena at 14 weeks of age. The challenge virus had been passaged three times on alveolar macrophages. At 42 days post-challenge (dpc), all pigs were euthanized by slow injection of an overdose of sodium pentobarbital 20% in the jugular vein.

The production of the reference immune sera against PRRSV strain Lena was performed at the IVI [25]. Briefly, three six-week-old SPF pigs (Swiss Landrace, in-house bred and reared) were infected with 5 × 10^5^ TCID_50_ Lena intranasally. Due to severe symptoms, one pig had to be euthanized seven days post-infection (dpi), while the other two pigs recovered from the infection and were kept until 52 dpi. Then, they were sacrificed, and we collected sera to establish the assay for detection of ADE antibodies.

### 2.2. Monocyte-Derived Macrophages

Peripheral blood mononuclear cells (PBMCs) were prepared with venous blood from 80–150 kg healthy pigs (Swiss Landrace). The pigs were held under specific pathogen-free (SPF) conditions. PBMCs were obtained by density centrifugation (Ficoll-Paque Plus, GE Healthcare Europe, Glattbrugg, Switzerland). From these, the monocytes were MACS^TM^-sorted on an LS-column (Miltenyi Biotec, Bergisch Gladbach, Germany) with the anti-CD172a monoclonal antibody (mAb), clone 74-22-15A (own production, hybridoma kindly provided by Prof. Armin Saalmüller, University of Vienna, Austria) and anti-mouse IgG-microbeads (Miltenyi Biotec). The purity of the sorted cells was 78–87%, depending on the experiment. The cells were cultured in phenol red-free Dulbecco’s modified Eagle’s medium (DMEM; Gibco, Thermo Fisher Scientific, Waltham, MA, USA), complemented with 10% fetal bovine serum (FBS; Thermo Fisher Scientific), and 20 U/mL porcine CSF-1 (own production [26]), plated at 0.5 × 10^6^ cells/mL and left to differentiate into monocyte-derived macrophages (MDMs) for 4 days at 39 °C, 5% CO_2_ [26].

### 2.3. Virus Preparation

Virus was grown on MDMs until a cytopathic effect (CPE) of 50% was reached. Then, the infected macrophages were frozen at −80 °C to lyse the cells. Subsequently, the cell lysate was centrifuged at 2000× *g* at 4 °C for 20 min to clarify the lysate and the viral titer was determined by titration on 4-day-old MDMs using IPMA with the mAb SR30-A (RTI, Brookings, SD, USA), directed against the PRRSV nucleocapsid (N). The tissue culture infective dose of 50% per mL (TCID_50_/mL) was calculated.

### 2.4. Quantification of Viremia and Viral Shedding In Vitro

**Figure 1:**
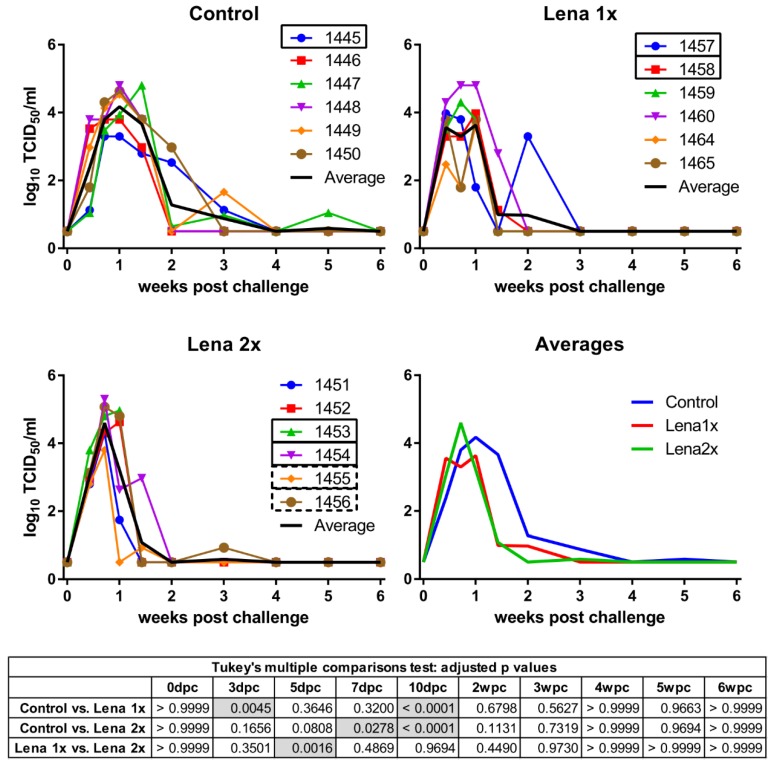
Viremia of Lena-vaccinated pigs following homologous challenge infection. Viremia was determined by titration on PAMs collected from porcine reproductive and respiratory syndrome virus (PRRSV)-negative pigs and IPMA. Pigs in the control group received two injections of PBS + adjuvant, eight and four weeks before the challenge. Group “Lena 1x” was vaccinated once with inactivated Lena, four weeks before the challenge. The group “Lena 2x” was vaccinated twice at eight and four weeks before the challenge. dpc—days post-challenge, wpc—weeks post-challenge. Numbers (1451–1465) represent the individual pigs. Solid rectangles around pig numbers mean they have been used in all ADE experiments, dashed rectangles mean that their sera have also been further analyzed, but not in all ADE experiments. The black line is the average of each group. Statistical analysis was performed with RM 2-way ANOVA, followed by Tukey’s multiple comparison test. Statistically significant values are highlighted in grey in the table under the graphs.

The viral quantification was performed by IPMA. A 10-fold serial dilution of serum (Figure 1) or cell culture supernatant (Figure 6) were incubated on 4-day-old MDMs for 48 h at 39 °C, 5% CO_2_. Then, the incubation media was removed, and the cells were fixed with 80% acetone for 10 min at room temperature (RT). The cells were washed with PBS, and for the detection of PRRSV-infected cells in the IPMA, the mAb 13E2 (own production, [27]; Figure 1) or mAb SR30-A (Figure 6) and horseradish-peroxidase conjugated polyclonal goat anti-mouse antibodies (Dako AS, Glostrup, Denmark) were used.

### 2.5. Quantification of Neutralizing Antibodies in Positive Control Sera

Neutralizing antibody titers of the positive control serum were determined on a porcine kidney cell line, PK15, which expresses CD163 and CD169 (“PK15^+/+^”) [28]. PK15^+/+^ cells, passage 10, were seeded at 2.5 × 10^5^ cells/mL in 96 well plates. Cells were cultured overnight at 37 °C, 5%CO_2_, in PK15^+/+^ medium (44% DMEM, 44% RPMI, 10% horse serum, 1% non-essential amino acids, and 1% Na-pyruvate (all Thermo Fisher Scientific)). For the rest of this experiment, 1000 U/mL penicillin/streptomycin (Thermo Fisher Scientific) were added to the medium. A serial 1:2 dilution of the immune sera in medium was performed in duplicate. An equal part of medium, containing 100 TCID_50_ (titer was determined on PK15^+/+^ cells), was added to the diluted immune sera. The virus–serum mixtures were allowed to form complexes for 30 min at 37 °C. Virus without immune sera was incubated alongside the virus–immune sera and served as a positive control for its continued infectivity. After this pre-incubation, the medium was removed from the PK15^+/+^ cells, and immune complexes or virus were allowed to infect the cells for 1 h at 37 °C, 5% CO_2_. The cells were washed once with warm PBS containing Mg^2+^ and Ca^2+^ (“PBS^+/+^”, Thermo Fisher Scientific) and fresh medium was added. After 48 h at 37 °C, 5%CO_2_, an IPMA assay was performed, as described above. The neutralizing titers were calculated as 50% neutralizing dose (ND50).

### 2.6. Antibody Responses during Vaccination Experiment

Serum samples were heat-inactivated for 30 min at 56 °C prior to testing. PRRSV-specific antibodies were detected by IPMA, as previously described [29,30]. Briefly, MARC-145 cells were seeded in 96-well plates, inoculated with PRRSV Lena virus and incubated for 24 h at 37 °C, 5% CO_2_. Then, the culture medium was removed and cells were washed with PBS and dried (1 h at 37 °C) After fixation with 4% paraformaldehyde (PFA, *w/v*; Polysciences, Warrington, PA, USA) for 10 min at RT, 1% H_2_O_2_ in methanol was added to the cells for 5 min, and cells were subsequently washed twice with PBS. Serial fourfold dilutions of the sera were added and incubated for 1 h at 37 °C. Plates were washed three times with PBS plus 1% Tween 80 and polyclonal goat anti-swine IgG/HRP (Dako; dilution 1:500) was added for 1 h (37 °C). The plates were washed again three times and 50 μL of a substrate solution (5% 3-amino-9-ethylcarbazole in 0.05 M acetate buffer, pH 5, with 0.05% H_2_O_2_) was added to each well and incubated for 20 min at RT. Then, the reaction was stopped by removing the substrate solution and replacing it with acetate buffer. Presence of anti-PRRSV antibodies was indicated by the deeply red stained cytoplasm of infected MARC-145 cells. The results were determined by examination with a microscope [29].

PRRSV-specific neutralizing antibodies from the vaccination challenge trial were titrated using a virus neutralization (VN) test on MARC-145 cells, as previously described [24].

### 2.7. ADE of Macrophage Infection

MDMs were prepared as described above, plated in 48-well plates, and left to differentiate for 72 h. We infected MDMs to achieve ca. 30% infected MDMs after 16 h incubation. It is noteworthy that in determining this MOI, one has to take into account that the virus will be incubated with the sera for 30 min at 37 °C, and a higher MOI is necessary to get 30% infected cells, compared to using the virus stock in absence of serum pre-incubation. Sera were heat-inactivated (56 °C, 30 min) and serially diluted in DMEM containing 1% FBS and 1000 U/mL Pen/Strep. Then, the sera were pre-incubated with MOI 0.02 of Lena for 30 min at 37 °C to allow immune complexes to form. One aliquot of the lowest dilution of each serum (1:30), was also incubated with cell lysate for mock controls of the respective immune serum. Directly after this pre-incubation, the medium was removed from the MDMs and replaced by the immune complex sera. Cells were infected in triplicates and infection was allowed for 1 h at 39 °C, 5% CO_2_. Then, the cells were washed twice with warm PBS^+/+^ and post-infection medium (DMEM, supplemented with 10% FBS, 20 U/mL M-CSF and 1000 U/mL Pen/Strep) was added. After 16 h at 39 °C, 5% CO_2_, the supernatants were collected and stored at −80 °C. The cells were harvested by 20 min incubation at RT with Accutase (Innovative Cell Technologies, San Diego, CA, USA). Then, cold PBS supplemented with 4.7 mM EDTA was added and the cells gently removed from the plates for flow cytometry staining.

### 2.8. Flow Cytometry

Briefly, cells were fixed with 4% PFA in PBS (Sigma-Aldrich). Then, the cells were washed and permeabilized with 0.1% saponin (AppliChem, Darmstadt, Germany) in PBS (*w/v*). For the detection of PRRSV, we used the mAb SR30-A for 20 min, followed by goat anti-mouse IgG_1_-AF647 (Molecular Probes, Thermo Fisher Scientific) for 15 min. Each reagent was diluted in 0.3% saponin in PBS (*w/v*) and incubations were done on ice. After a final washing step, the cells were resuspended in CellWash^®^ (Becton Dickinson, Allschwil, Switzerland) and acquired with a FACSCanto (Becton Dickinson). The data were analyzed with the FlowJo software (Tree Star, Ashland, OR, USA). Doublets were excluded in FSC A/W and SSC A/W, and then a gate was set on the big cells in FSC-A/SSC-A, to exclude debris and dead cells.

### 2.9. ELISA

Cytokines were measured in the supernatants of infected macrophages. The following cytokines were tested: porcine IL-1β/IL-1F2 (Kit: DY681), IL-6 (Kit: DY686), IL-10 (Kit: DY693B), IL-12/IL-23 p40 (Kit: DY912), and TNF (Kit: DY690B; all kits were obtained from R&D Systems, Minneapolis, MN, USA).

### 2.10. Statistical Analysis

Statistical analyses were performed with repeated measures (RM) 2-way ANOVA, followed by Tukey’s post hoc analysis. *p* < 0.05 was considered to be statistically significant. If a different statistical test was used, it is mentioned in the figure legends. Statistical analysis was performed with Prism 6 software (GraphPad Software, San Diego, CA, USA).

## 3. Results

### 3.1. Viremia and PRRSV Neutralization Titers after Infection of Naïve Animals for Generation of the Reference Immune Serum

After the infection of three pigs with PRRSV Lena, the animals developed a viremia within 24 h of infection that peaked at 5–6 dpi. Due to the severity of clinical symptoms, which included the inability to get up, one of the animals had to be euthanized at eight dpi, while the other two animals slowly gained control over the virus infection. They were symptom-free after 17–18 dpi, although their viremia lasted until 28 or 35 dpi. The surviving pigs developed detectable neutralizing antibody titers by 21 or 28 dpi. At 52 dpi, they had ND50 titers of 1:11 for pig 2 and 1:17 for pig 3. The dynamics of the viremia have previously been published [25]. The serum of pig 3 served as “positive control serum” in the presented experiments (Figures 5 and 6).

### 3.2. Vaccination with an Inactivated Virus and Homologous Challenge

We have recently developed a novel inactivated vaccine against PRRSV-1, subtype 1 strains, that showed promising protective results [31]. At the time of these experiments, no vaccine was yet available against the highly pathogenic strain Lena and, consequently, we tested the efficacy of a similarly prepared vaccine against Lena. Conventionally raised pigs were vaccinated once (Lena 1x) or twice (Lena 2x) with inactivated Lena virus and subsequently challenged with PAM-grown Lena. All of the animals in the vaccination trial developed viremia (Figure 1). The pigs from the Lena 1x group developed a significantly higher viremia than the control group early during the infection (at 3dpc), but never peaked as high as the other groups. Meanwhile, the animals that had been vaccinated twice were able to reduce their viral titers one week before the control group, but they also peaked significantly higher in viremia than the Lena 1x pigs, and one pig had to be euthanized due to the severity of clinical symptoms. Nevertheless, after 10 dpc, both vaccinated groups had a significantly reduced viremia compared to the unvaccinated pigs; the Lena 2x group even already at 7 dpc. All surviving pigs were able to eliminate the virus from their blood, though each group had at least one animal that experienced a rebound of viremia.

### 3.3. Body Temperature and Respiratory Disease Score

**Figure 2:**
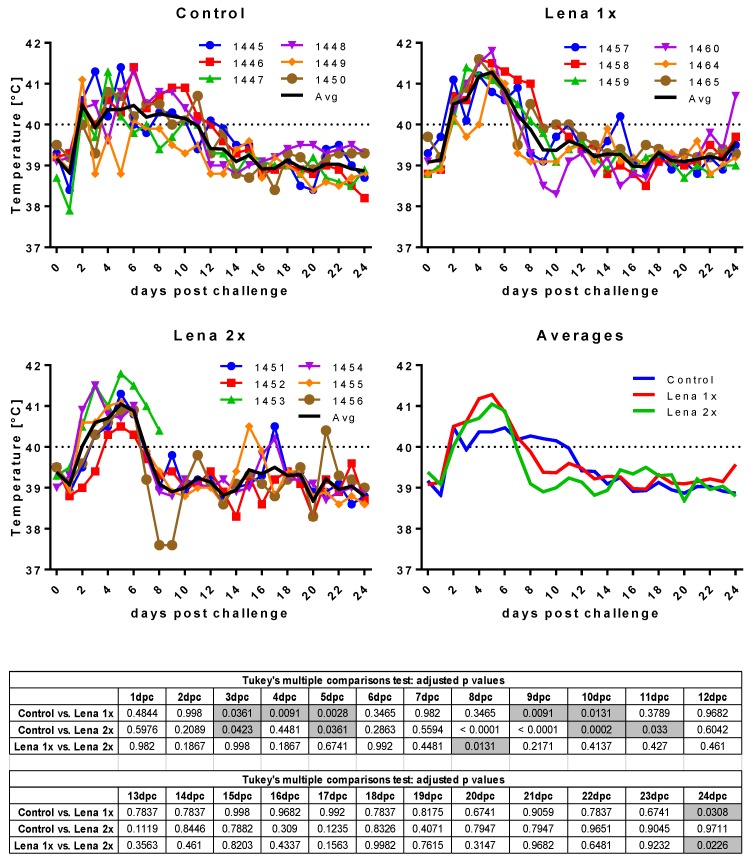
Body temperature after challenge. Control, Lena 1x, and Lena 2x: Colored lines represent individual animals and thick black lines represent the mean value for each group. Temperature >40 °C was considered as fever (dotted line on upper panel). dpc—days post-challenge. Statistical analysis was performed with an ordinary 2-way ANOVA, followed by Tukey’s multiple comparison test. Statistically significant values are highlighted in grey in the table under the graphs.

**Figure 3:**
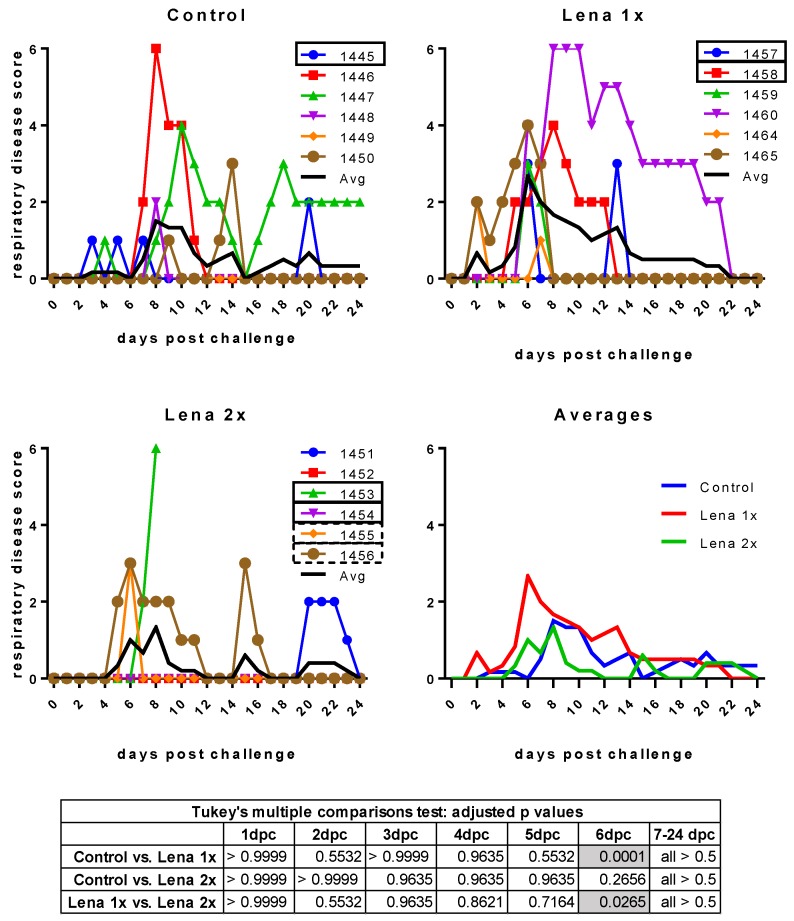
Respiratory disease scores after challenge. Control, Lena 1x, and Lena 2x: Colored lines represent individual animals and thick black lines represent the mean value for each group. Scores: 0 = normal; 1 = mild dyspnea and/or tachypnea when stressed; 2 = mild dyspnea and/or tachypnea at rest; 3 = moderate dyspnea and/or tachypnea when stressed; 4 = moderate dyspnea and/or tachypnea at rest; 5 = severe dyspnea and/or tachypnea when stressed; 6 = severe dyspnea and/or tachypnea at rest. dpc—days post-challenge. Statistical analysis was performed with an ordinary 2-way ANOVA, followed by Tukey’s multiple comparison test. Statistically significant values are highlighted in grey in the table under the graphs.

Surprisingly, despite the presence of neutralizing antibodies, all animals developed clinical signs of infection upon challenge with Lena (Figure 2 and Figure 3). Even more unexpectedly, four piglets in the vaccinated groups showed severe symptoms (fever up to 41.8 °C, hypothermia down to 37.6 °C, tremor, convulsions, diarrhea). These symptoms were so severe in the case of one pig in the “Lena 2x” group (pig #1453) that it had to be euthanized at 7 dpc.

The respiratory symptoms were scored as follows: 0 = normal breathing (20–40 breaths per minute); 1 = mild dyspnea and/or tachypnea (40–60 breaths per minute) when stressed by holding the pig for 45 s; 2 = mild dyspnea and/or tachypnea (40–60 breaths per minute) at rest; 3 = moderate dyspnea and/or tachypnea (60–80 breaths per minute) when stressed; 4 = moderate dyspnea and/or tachypnea (60–80 breaths per minute) at rest; 5 = severe dyspnea and/or tachypnea (>80 breaths per minute) when stressed; 6 = severe dyspnea and/or tachypnea (>80 breaths per minute) at rest.

### 3.4. PRRSV-Specific and PRRSV-Neutralizing Antibody Titers

**Figure 4:**
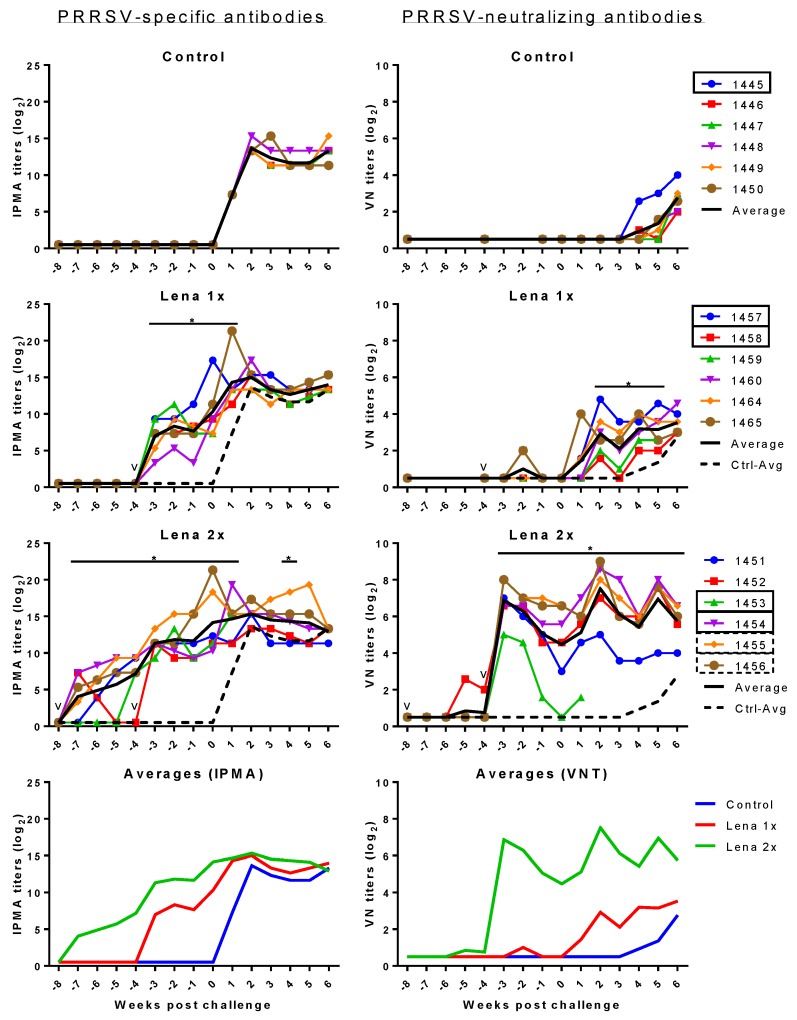
PRRSV-specific IPMA (left) and VN (right) antibodies before and after challenge 10^5^ TCID_50_ PRRSV Lena. Control pigs received two injections of PBS in adjuvant, at eight and four weeks before the challenge. Group “Lena 1x” was vaccinated once, four weeks before the challenge. Group “Lena 2x” received a prime/booster vaccination at eight and four weeks before the challenge. PRRSV-specific antibodies were detected by IPMA. PRRSV-neutralizing antibodies were detected by a virus neutralization assay (VNT). Colored lines with symbols represent individual pigs. Solid black lines represent the average of each group and dashed black lines represent the average of the control group. v—vaccination; numbers represent individual pig numbers. Solid rectangles around pig numbers mean they have been used in all ADE experiments, dashed rectangles mean their sera have also been further analyzed, but not in all ADE experiments. Statistical analysis was performed with RM 2-way ANOVA, followed by Tukey’s multiple comparison test. The comparison was: Lena 1x vs. Control, and Lena 2x vs. Control. *p* < 0.05 was considered statistically significant and is represented by *.

All animals in the Lena 1x group developed PRRSV antibodies (Figure 4 left panels), but neutralizing antibodies were not found or only transiently detectable (Figure 4 right panels). After the challenge, all animals in this group developed neutralizing antibodies. The animals in the Lena 2x group developed high titers of neutralizing antibodies after the booster, but the titer of one of these animals (pig #1453) declined before the challenge. This pig had to be euthanized because of the severity of its symptoms upon challenge.

Over the entire course of the experiment, the titers of neutralizing antibodies in the booster-vaccinated group remained high. Nonetheless, the presence of these neutralizing antibodies did not appear to have a beneficial effect on the level of the viremia (Figure 1).

### 3.5. ADE of Macrophage Infection

**Figure 5:**
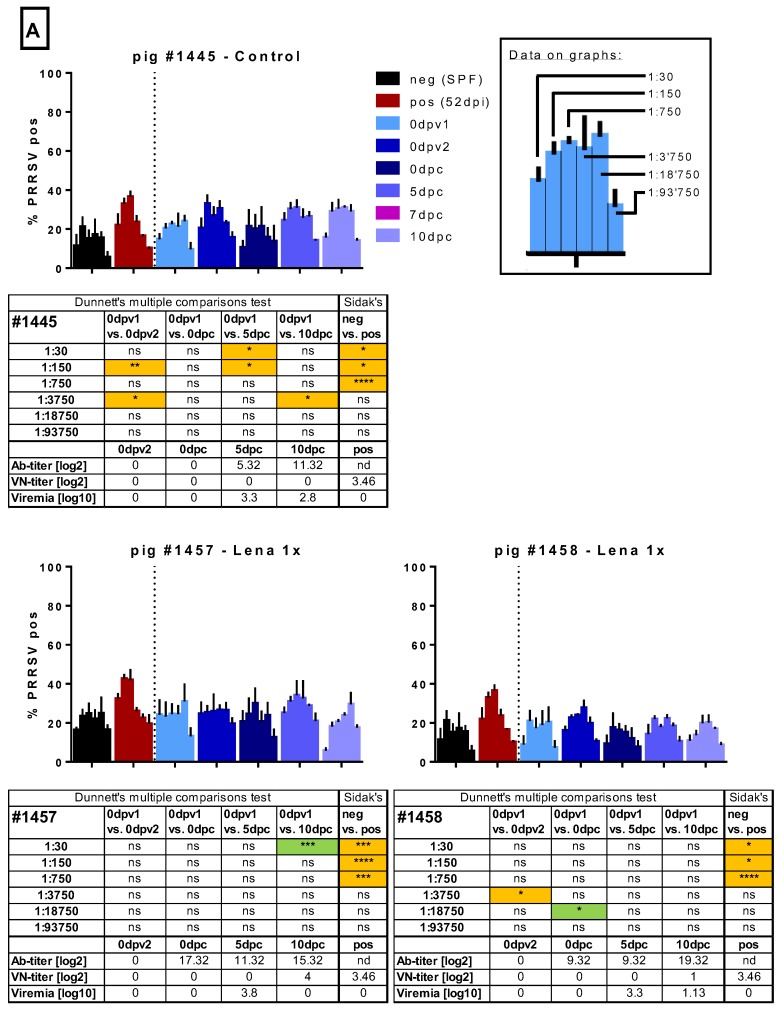

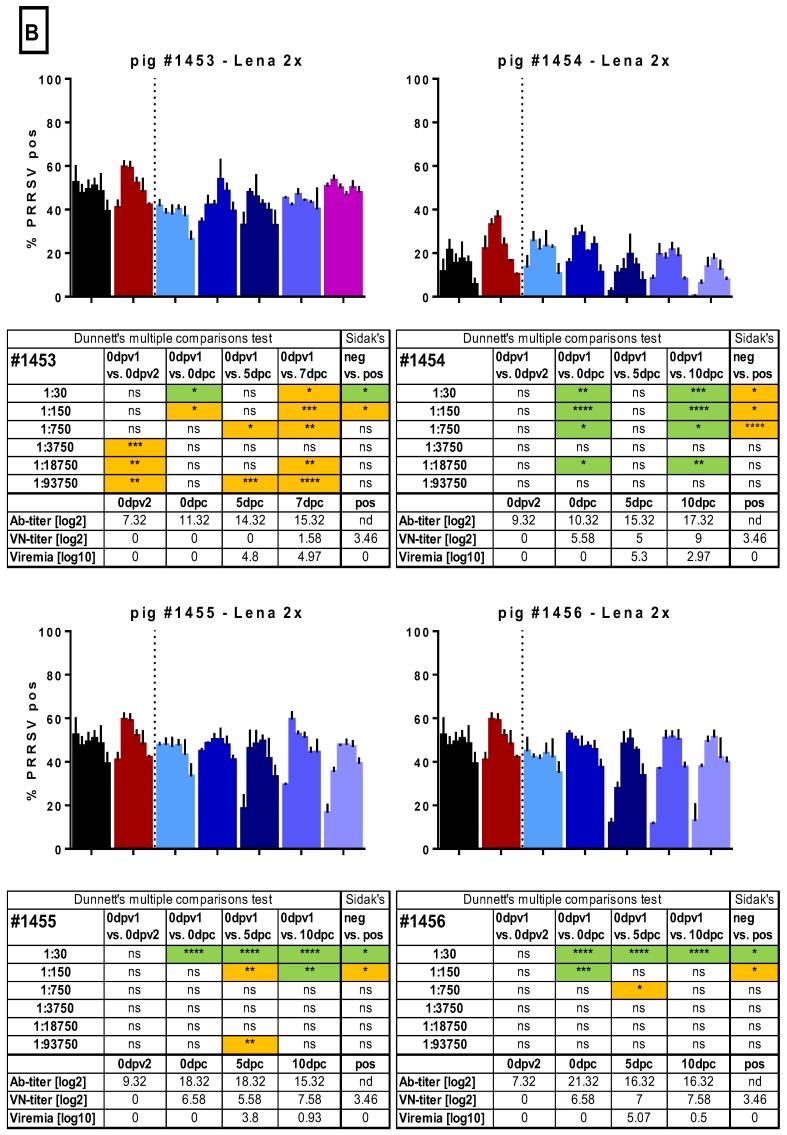
Macrophage infection in the presence of immune sera. Sera of several randomly selected pigs from the different groups were tested for their ability to enhance the infection of monocyte-derived macrophages (MDMs). Serial dilutions (1:30 to 1:93,750) of the sera were pre-incubated with Lena. Then, MDMs were infected in triplicates and tested PRRSV N-protein after 16 h by flow cytometry. In A, the results for pig #1445, from the control group, which received 2 injections of PBS + adjuvant, as well as the results for pigs #1457 and #1458, from the “Lena 1x” group, which were vaccinated once, is shown. In B. the results for pigs #1453–1456, from the “Lena 2x” group, which were vaccinated twice, is shown. Bars show mean with SD. dpv—days post-vaccination, dpc—days post-challenge, dpi—days post-infection; 0dpv1 = eight weeks before challenge, 0dpv2 = four weeks before challenge. Statistical analysis was performed with ordinary 2-way ANOVA, followed by Dunnett’s multiple comparison test or Sidak’s post hoc test, as indicated. Green highlighting indicates a neutralizing effect, while orange highlighting indicates an enhancing effect. Symbols: * = *p* < 0.05, ** = *p* < 0.01, *** = *p* < 0.001, **** = *p* < 0.0001.

In the next step, we tested whether the sera would influence the infection of MDMs. To this end, we chose an infectious dose of Lena that would yield between 25% and 50% infected cells after 16 h of incubation. To avoid the impact of inter-experiment variation, all sera of one pig, including the pre-vaccination time point, were tested together with a defined negative control (serum from an SPF pig) and a selected potentially “positive” control (Lena-infected pigs with neutralizing antibodies). Virus pre-incubated with the positive control serum resulted in more infected cells at a dilution of 1:150 in 7/7 experiments run in triplicate. In some, but not all, experiments with the same serum, ADE was also found at the 1:750 dilution (Figure 5). This constitutes a dilution of factor 13 to 68 beyond the neutralizing titer. The dilution of 1:30 was enhancing the macrophage infection in two of the three shown experiments, and once acting in a neutralizing manner. While these experiments indicate that ADE could be happening with PRRSV immune sera, the effect was mild and varied considerably between experiments.

In the sera from the randomly selected control animal #1445, we occasionally found evidence of ADE, but this was also observed before challenge and was not consistently found at particular dilutions nor did it appear to be titratable (Figure 5). In the two sera from the randomly selected Lena 1x group, we did not find any consistent evidence for ADE. The serum with neutralizing antibodies at 10 dpc had a neutralizing activity also on MDMs (Figure 5).

From the Lena 2x group, we selected four sera randomly to test in the ADE assay (Figure 5). Pig #1453 appeared to have developed detectable ADE activity at very high serum dilutions after the first vaccination, but this was not found with the sera from the other pigs nor with the sera from the Lena 1x group. Furthermore, with the sera collected later, apparent ADE was again found at the two time points post-challenge. However, this activity did not follow a titration-dependent increase and decline, indicating that it was not solely dependent on one factor. The observation that this was not found with the other sera from this group led us to the conclusion that no ADE activity was found in the Lena 2x group. However, with the other three pigs in this group, we found that sera with neutralizing activity reduced the infectivity of MDMs in most cases (Figure 5).

**Figure 6:**
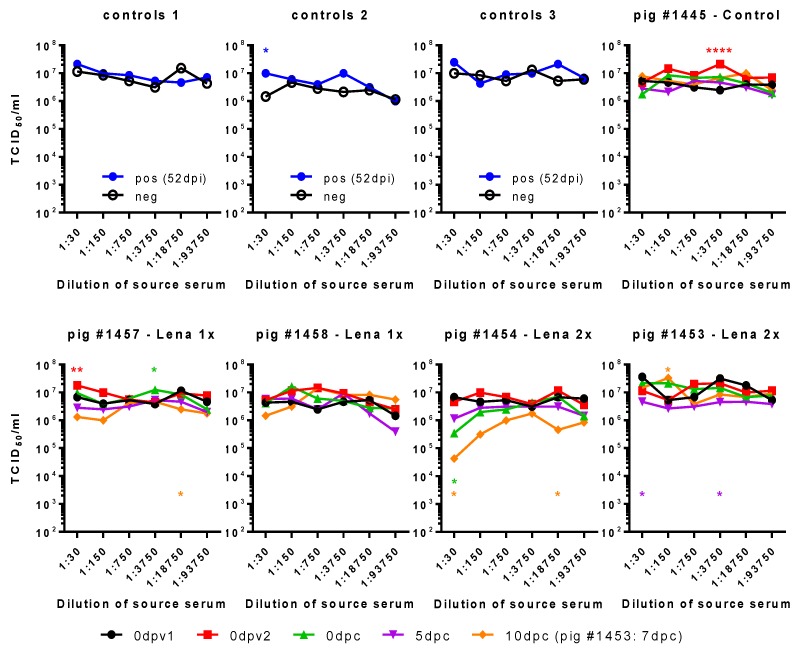
Impact of sera on virus titers in MDM cultures. Serially diluted immune sera were pre-incubated with Lena. Then, MDMs were infected with these immune complex mixtures, washed, and incubated for a total of 16 h. The supernatants were titrated in triplicates. Controls 1–3 show the viral titers when PRRSV-Ab-negative serum (neg) or PRRSV-immune serum (pos, from 52 dpi) were used as immune sera. Pig #1445, from the control group, had not been immunized before challenge. Pigs #1457 and #1458, from the “Lena 1x” group, were vaccinated once with BEI-inactivated Lena + adjuvant, and pig #1454, from the “Lena 2x” group, was vaccinated twice with the same vaccine. dpv—days post-vaccination, dpc—days post-challenge, dpi—days post-infection; 0dpv1 = eight weeks before challenge, 0dpv2 = four weeks before challenge. Statistical analysis was performed with ordinary 2-way ANOVA, followed by Tukey’s multiple comparison test. Within each dilution, the sera from different time points were compared with the “initial serum” (neg or 0dpv1). Symbols: * = *p* < 0.05, ** = *p* < 0.01, *** = *p* < 0.001, **** = *p* < 0.0001. Color of the asterisks denote to which serum they belong.

In conclusion, these tests did not permit us to identify ADE as a possible cause of enhanced disease. Furthermore, the MDM assay system based on nucleoprotein measurement of infectivity was not robust in terms of its reproducibility. We, therefore, tested if viral progeny from these cultures was influenced by the different sera (Figure 6). In three independent cultures, the positive control serum induced only once increased viral shedding at a 1:30 dilution. Curiously, we found that, indeed, the serum of pig #1445 of 0dpv2 led to increased shedding of virus. It is the same serum and dilution that had also led to a higher percentage of infected MDMs, while the pig had not been exposed to any vaccine or virus, yet. We also observed two small, yet statistically significant, increases for pig #1457 with the sera 0dpv2, 1:30, and 0dpc, 1:3750, and a reduced titer for 10dpc, 1:18,750, all of which did not correspond to any increase or reduction in infection of MDMs. None of the SNs of the sera tested for pig #1458 differed significantly from the control. For pig #1454 from the Lena 2x group, we saw a clear reduction in the viral titers at 1:30 10 dpc.

We also checked the presence of cytokines in the supernatants that were derived with the sera of 0 dpc and 5 dpc, dilutions 1:30 to 1:750, of the pigs #1445, #1458, #1453, and #1454; but were unable to detect any induction of IL-1β, IL-6, IL-10, IL-12, or TNF (data not shown).

In summary, neither the neutralizing effect of the sera nor the slight enhancement of MDM-infection translated into consistently strong effects regarding the viral shedding or any effect on cytokine release.

## 4. Discussion

PRRSV mutates frequently, and because of a generally restricted heterologous protection, quick development of protective vaccines is of great importance to the pig production community. Immunization with an inactivated field strain could, in principle, serve that purpose; by employing a high antigen payload and a potent adjuvant, we previously demonstrated that such vaccines can have a protective value [24,31]. This was the basis for employing such a vaccine approach for Lena, which is a particularly highly pathogenic (HP) PRRSV strain that emerged recently in Eastern Europe [23] and has afflicted it since. Therefore, we vaccinated two groups of pigs either once or twice with BEI-inactivated Lena. While the vaccination was found to be successful in terms of the ability to induce anti-PRRSV antibodies after a single injection or even to induce neutralizing antibodies after the prime/boost vaccination, the vaccinations were disappointing for their protective capacity. In fact, there were even signs of enhanced disease in some of the vaccinated animals. This lack of protection in the presence of neutralizing antibodies is in direct conflict with several previous reports, where the adoptive transfer to reach neutralizing antibody serum levels was protective [14,32,33,34]. It also contradicts many studies which observed a positive correlation between neutralizing antibodies and control of viremia [20,21,22]. This discrepancy may be due to strain-specific characteristics or unknown environmental factors affecting the vaccinated pigs. Nevertheless, it is not possible to attribute this to the inactivated vaccine as this has been previously found to be efficacious with other strains of PRRSV [31,35].

Due to this unexpected outcome, we investigated the possible involvement of ADE antibodies in the deteriorative clinical outcome of this vaccination trial. We tested the sera for the capacity to mediate ADE in porcine MDMs, a natural target cell of PRRSV, which constitutively expresses CD16 and CD64. The results demonstrate that immune sera that neutralized virions on a cell line assay also neutralized the infection of the MDMs. This neutralizing effect was only lost at a dilution of 1:150/1:750. Beyond this dilution, we only rarely observed a very slight enhancement of MDM infection. We did, however, also detect the occasional statistical significance in enhancement or neutralization of infection for sera that were still naïve or had no detectable levels of neutralizing antibodies, respectively. Nevertheless, the enhancing effects were mild and often did not titrate. It is important to bear in mind that PRRSV infectivity is influenced by many factors in the serum, not only by antibodies. Furthermore, statistical significance and biological significance are not synonymous.

Alternative explanations for the enhanced disease observed in the vaccinated groups could be that FcRs on other cells, such as monocytes and dendritic cells, could be targeted by the immune complexes, resulting in infection and possibly inflammatory reactions. Furthermore, an involvement of the complement system or vaccine-primed T cells needs to be considered. Complement has been proposed to play a role for ADE of West Nile virus (WNV) [36] and HIV [37,38]. In our setup, the heat-inactivation of the sera prevented us from testing such effects. Recently, Bordet et al. showed a strain-specific increased secretion of IFNγ by polarized T helper 1 (Th1) cells in response to the Lena strain [39]. It is possible that the vaccination(s) in our experiment primed these T cells, leading to enhanced inflammatory responses, which exacerbated the clinical symptoms.

## 5. Conclusions

We conclude that ADE of macrophage infection mediated solely by antibodies is not a feature in this vaccination challenge experiment and cannot explain the lack of protection or even enhanced disease following the homologous challenge with the subtype 3 PRRSV-1 strain Lena. Also, sera from naturally infected pigs with the same strain of PRRSV had no or only a weak ADE activity. Furthermore, there are now a total of four experimental studies demonstrating the protective role of antibodies against PRRSV, and that this effect is correlated to neutralizing activity [14,32,33,34]. Therefore, future vaccine design should continue to focus on the induction of broadly neutralizing antibodies. With respect to the employment of inactivated vaccines, the present work also demonstrates that extrapolations from one PRRSV-1 strain to another can be risky. While such vaccines, if prepared appropriately, can be protective [24,31], the outcome with the Lena strain was detrimental despite induction of neutralizing antibodies. This is adding one more piece to the puzzling immunology of PRRS.

## References

[1] Cafruny W.A., Plagemann P.G. (1982). Immune response to lactate dehydrogenase-elevating virus: Serologically specific rabbit neutralizing antibody to the virus. Infect. Immun..

[2] Takeda A., Tuazon C.U., Ennis F.A. (1988). Antibody-enhanced infection by hiv-1 via fc receptor-mediated entry. Science.

[3] Homsy J., Meyer M., Tateno M., Clarkson S., Levy J.A. (1989). The fc and not cd4 receptor mediates antibody enhancement of hiv infection in human cells. Science.

[4] Halstead S.B., Porterfield J.S., O’Rourke E.J. (1980). Enhancement of dengue virus infection in monocytes by flavivirus antisera. Am. J. Trop. Med. Hyg..

[5] Halstead S.B. (2014). Dengue antibody-dependent enhancement: Knowns and unknowns. Microbiol. Spectr..

[6] Garcia-Nicolas O., Ricklin M.E., Liniger M., Vielle N.J., Python S., Souque P., Charneau P., Summerfield A. (2017). A japanese encephalitis virus vaccine inducing antibodies strongly enhancing in vitro infection is protective in pigs. Viruses.

[7] Mason P.W., Baxt B., Brown F., Harber J., Murdin A., Wimmer E. (1993). Antibody-complexed foot-and-mouth disease virus, but not poliovirus, can infect normally insusceptible cells via the fc receptor. Virology.

[8] Baxt B., Mason P.W. (1995). Foot-and-mouth disease virus undergoes restricted replication in macrophage cell cultures following fc receptor-mediated adsorption. Virology.

[9] Lannes N., Python S., Summerfield A. (2012). Interplay of foot-and-mouth disease virus, antibodies and plasmacytoid dendritic cells: Virus opsonization under non-neutralizing conditions results in enhanced interferon-alpha responses. Vet. Res..

[10] Boonnak K., Dambach K.M., Donofrio G.C., Tassaneetrithep B., Marovich M.A. (2011). Cell type specificity and host genetic polymorphisms influence antibody-dependent enhancement of dengue virus infection. J. Virol..

[11] Kou Z., Lim J.Y., Beltramello M., Quinn M., Chen H., Liu S., Martinez-Sobrido L., Diamond M.S., Schlesinger J.J., de Silva A. (2011). Human antibodies against dengue enhance dengue viral infectivity without suppressing type i interferon secretion in primary human monocytes. Virology.

[12] Lidbury B.A., Mahalingam S. (2000). Specific ablation of antiviral gene expression in macrophages by antibody-dependent enhancement of ross river virus infection. J. Virol..

[13] Suhrbier A., La Linn M. (2003). Suppression of antiviral responses by antibody-dependent enhancement of macrophage infection. Trends Immunol..

[14] Yoon K.J., Wu L.L., Zimmerman J.J., Hill H.T., Platt K.B. (1996). Antibody-dependent enhancement (ade) of porcine reproductive and respiratory syndrome virus (prrsv) infection in pigs. Viral Immunol..

[15] Yoon K.J., Wu L.L., Zimmerman J.J., Platt K.B. (1997). Field isolates of porcine reproductive and respiratory syndrome virus (prrsv) vary in their susceptibility to antibody dependent enhancement (ade) of infection. Vet. Microbiol..

[16] Qiao S., Jiang Z., Tian X., Wang R., Xing G., Wan B., Bao D., Liu Y., Hao H., Guo J. (2011). Porcine fcgammariib mediates enhancement of porcine reproductive and respiratory syndrome virus (PRRSV) infection. PLoS ONE.

[17] Gu W., Guo L., Yu H., Niu J., Huang M., Luo X., Li R., Tian Z., Feng L., Wang Y. (2015). Involvement of cd16 in antibody-dependent enhancement of porcine reproductive and respiratory syndrome virus infection. J. Gen. Virol..

[18] Shi P., Su Y., Li Y., Zhang L., Lu D., Li R., Zhang L., Huang J. (2019). The alternatively spliced porcine fcgammari regulated prrsv-ade infection and proinflammatory cytokine production. Dev. Comp. Immunol..

[19] Cancel-Tirado S.M., Evans R.B., Yoon K.J. (2004). Monoclonal antibody analysis of porcine reproductive and respiratory syndrome virus epitopes associated with antibody-dependent enhancement and neutralization of virus infection. Vet. Immunol. Immunopathol..

[20] Loving C.L., Osorio F.A., Murtaugh M.P., Zuckermann F.A. (2015). Innate and adaptive immunity against porcine reproductive and respiratory syndrome virus. Vet. Immunol. Immunopathol..

[21] Rahe M.C., Murtaugh M.P. (2017). Mechanisms of adaptive immunity to porcine reproductive and respiratory syndrome virus. Viruses.

[22] Rahe M.C., Murtaugh M.P. (2017). Effector mechanisms of humoral immunity to porcine reproductive and respiratory syndrome virus. Vet. Immunol. Immunopathol..

[23] Karniychuk U.U., Geldhof M., Vanhee M., Van Doorsselaere J., Saveleva T.A., Nauwynck H.J. (2010). Pathogenesis and antigenic characterization of a new east european subtype 3 porcine reproductive and respiratory syndrome virus isolate. BMC Vet. Res..

[24] Vanhee M., Delputte P.L., Delrue I., Geldhof M.F., Nauwynck H.J. (2009). Development of an experimental inactivated prrsv vaccine that induces virus-neutralizing antibodies. Vet. Res..

[25] Garcia-Nicolas O., Auray G., Sautter C.A., Rappe J.C., McCullough K.C., Ruggli N., Summerfield A. (2016). Sensing of porcine reproductive and respiratory syndrome virus-infected macrophages by plasmacytoid dendritic cells. Front. Microbiol..

[26] Sautter C.A., Auray G., Python S., Liniger M., Summerfield A. (2018). Phenotypic and functional modulations of porcine macrophages by interferons and interleukin-4. Dev. Comp. Immunol..

[27] Van Breedam W., Costers S., Vanhee M., Gagnon C.A., Rodriguez-Gomez I.M., Geldhof M., Verbeeck M., Van Doorsselaere J., Karniychuk U., Nauwynck H.J. (2011). Porcine reproductive and respiratory syndrome virus (prrsv)-specific mabs: Supporting diagnostics and providing new insights into the antigenic properties of the virus. Vet. Immunol. Immunopathol..

[28] Delrue I., Van Gorp H., Van Doorsselaere J., Delputte P.L., Nauwynck H.J. (2010). Susceptible cell lines for the production of porcine reproductive and respiratory syndrome virus by stable transfection of sialoadhesin and cd163. BMC Biotechnol..

[29] Decorte I., Van Breedam W., Van der Stede Y., Nauwynck H.J., De Regge N., Cay A.B. (2014). Detection of total and prrsv-specific antibodies in oral fluids collected with different rope types from prrsv-vaccinated and experimentally infected pigs. BMC Vet. Res..

[30] Labarque G.G., Nauwynck H.J., Van Reeth K., Pensaert M.B. (2000). Effect of cellular changes and onset of humoral immunity on the replication of porcine reproductive and respiratory syndrome virus in the lungs of pigs. J. Gen. Virol..

[31] Geldhof M.F., Vanhee M., Van Breedam W., Van Doorsselaere J., Karniychuk U.U., Nauwynck H.J. (2012). Comparison of the efficacy of autogenous inactivated porcine reproductive and respiratory syndrome virus (prrsv) vaccines with that of commercial vaccines against homologous and heterologous challenges. BMC Vet. Res..

[32] Osorio F.A., Galeota J.A., Nelson E., Brodersen B., Doster A., Wills R., Zuckermann F., Laegreid W.W. (2002). Passive transfer of virus-specific antibodies confers protection against reproductive failure induced by a virulent strain of porcine reproductive and respiratory syndrome virus and establishes sterilizing immunity. Virology.

[33] Lopez O.J., Oliveira M.F., Garcia E.A., Kwon B.J., Doster A., Osorio F.A. (2007). Protection against porcine reproductive and respiratory syndrome virus (prrsv) infection through passive transfer of prrsv-neutralizing antibodies is dose dependent. Clin. Vaccine Immunol..

[34] Robinson S.R., Rahe M.C., Gray D.K., Martins K.V., Murtaugh M.P. (2018). Porcine reproductive and respiratory syndrome virus neutralizing antibodies provide in vivo cross-protection to prrsv1 and prrsv2 viral challenge. Virus Res..

[35] Geldhof M.F., Van Breedam W., De Jong E., Lopez Rodriguez A., Karniychuk U.U., Vanhee M., Van Doorsselaere J., Maes D., Nauwynck H.J. (2013). Antibody response and maternal immunity upon boosting prrsv-immune sows with experimental farm-specific and commercial prrsv vaccines. Vet. Microbiol..

[36] Cardosa M.J., Porterfield J.S., Gordon S. (1983). Complement receptor mediates enhanced flavivirus replication in macrophages. J. Exp. Med..

[37] Prohaszka Z., Nemes J., Hidvegi T., Toth F.D., Kerekes K., Erdei A., Szabo J., Ujhelyi E., Thielens N., Dierich M.P. (1997). Two parallel routes of the complement-mediated antibody-dependent enhancement of hiv-1 infection. AIDS.

[38] Robinson W.E., Montefiori D.C., Mitchell W.M. (1990). Complement-mediated antibody-dependent enhancement of hiv-1 infection requires cd4 and complement receptors. Virology.

[39] Bordet E., Blanc F., Tiret M., Crisci E., Bouguyon E., Renson P., Maisonnasse P., Bourge M., Leplat J.J., Giuffra E. (2018). Porcine reproductive and respiratory syndrome virus type 1.3 lena triggers conventional dendritic cells 1 activation and t helper 1 immune response without infecting dendritic cells. Front. Immunol..

